# Puerperal Fever

**Published:** 1823-03-01

**Authors:** 


### PUERPERAL FEVER.

1. *A Treatise on the Epidemic Puerperal Fever, as it prevailed in Edinburgh in* 1821-22. *To which is added an Appendix, containing the Essay of the late Dr. Gordon on the Puerperal Fever of Aberdeen in* 1789-90-91-92. By William Campbell, M. D. Fellow of the Royal College of Surgeons; one of the Medical Officers of the Royal Public Dispensary, Lecturer on Midwifery. 8vo, pp. 400. Edinburgh and London, 1822.

2. *A Treatise on the Disease termed Puerperal Fever; illustrated by numerous Cases and Dissections.* By John Mackintosh, M.D. Octavo, pp. 323. Edinburgh and London, 1822.

This fatal scourge—this "morbus lethalis," as Hippocrates describes it, "ex quo paucæ effugere possunt," has been assuming one of its worst forms, the *epidemic*, and carrying devastation through the lower orders of the Scottish metropolis, notwithstanding the great strength of the doctorate in that quarter.

Although the pathology of the disease termed puerperal fever is pretty unanimously agreed upon on this side of the Tweed, yet there is still some little discrepancy here respecting the treatment. Beyond the Tweed all is discord in regard both to pathology and treatment. An eminent obstetrical teacher in the intellectual city has marshalled under his banners a considerable host of practitioners who declare war against the lancet, and maintain that real puerperal fever is a disease differing, in some mysterious manner, from puerperal peritonitis, with which, they aver, it is confounded by the other party. Many active and intelligent practitioners, however, guided by the light of pathology, have dared to dissent from the dictum of the medical autocrat and his proselytes, and to affirm that the fever which, in all ages, has proved so fatal to parturient females is truly inflammatory, while the seat of the inflammation is the peritoneum or sexual organs. Among this class are to be ranked the two authors before us, who have enriched the medical and pathological literature of their country by two publications of very considerable merit. As puerperal fever has not yet occupied an article in the present series of the journal, we shall take this opportunity of entering pretty fully into the subject, and of presenting to our readers as much as possible of the valuable matters contained in the volumes now on our table. It is to be regretted, we think, that the two gentlemen in question did not content themselves with giving a full yet explicit account of the epidemic that passed under their review, without deeming it necessary, each to compose a treatise on the disease, by searching the records of the past, and adding the evidence of the dead and the living to their own observations. It is in this way that books are sometimes needlessly multiplied, and documents rendered complicated and in some degree confused.

In each work we have a historical introduction; that of Dr. Mackintosh being the more rambling and diffuse of the two, occupying 157 pages. We shall cull a slight sketch from these chapters.

Hippocrates has described puerperal fever with more distinctness than most other diseases. He has also, as we before said, characterized it as almost always fatal. Historical traces of the complaint may be found in most of the ancients, from the father of physic downwards. We shall not dwell on any of the ancient records, but come at once to a comparatively recent period.

Strother, about the year 1716, gave the disease the term puerperal fever, and considered it an inflammation of the uterus. Dr. William Hunter asserted that puerperal fever caused the death of two-thirds of those women who died in child-bed.* Hulme, who wrote an excellent treatise on the disease, declared that it was as much to be dreaded as the plague. Denman looked upon it in nearly the same light; and Clarke observed that the most experienced practitioners were staggered at the fatality and embarrassed in the treatment of the disease. In 1770, when it prevailed in London, 19 out of 63 who were delivered in the Westminster new Lying-in-Hospital had the disease, and of these 19, thirteen died. Between the years 1789 and 1792 it prevailed, more or less, in Aberdeen, and gave occasion to the excellent little tract of Dr. Gordon, who states that out of 77 patients he lost 28—the great mortality prevailing at first, when he bled sparingly; for out of the first 27 cases, 23 died—but of the

---

* This great physician, says Dr. Campbell, was in the habit of informing his pupils that of 32 patients who were attacked with the disease during two months, only one recovered. "We tried," says he, "various methods. One woman we took from the beginning and bled her, and she died. To a third we gave confect. aromat. and other cordials and stimuli, and she died. In another we gave cooling medicines, and she also died." *MS. Lectures quoted by Dr. Campbell.*

remaining 50 cases, when he came to adopt an energetic practice, by bleeding boldly and early, he only lost five patients. In Home's clinical experiments, we are informed that all who were attacked with puerperal fever in the lying-in wards of the Royal Infirmary, died! Dr. Mackintosh is sorry to add, "that in the Lying-in-hospital, as well as out of it, the practice adopted has not met with much better success."

"Such" says Dr. Campbell, "was the mortality attending this disease in the practice of three successive professors of midwifery in a celebrated northern university, that the first pronounced it to be incurable, the second declared that we were not only ignorant of the nature of the disease, but equally so of any remedy calculated to afford relief; and the third is so satisfied with the justness of the observations of his predecessors, that to this day, when cases of the disease are related to have been cured, he cannot allow them to have been examples of puerperal fever, but of some other affection confounded with it," P. 11.

To the opinions of the above-mentioned eminent characters, Dr. Campbell's experience enables him to add, that there is no disease of more fatal tendency than the one under consideration—"none in which Nature unassisted can accomplish less, or art more." If neglected in the beginning, he acknowledges that the physician, however great his talents, can only be a melancholy spectator of mischief which he cannot remedy.

"I beg the reader," says Dr. Campbell, "will bear in mind, that I consider myself equally justified in asserting, that the puerperal fever, *if detected early, and treated upon principle*, from the commencement, admits of being cured with as much certainty as other diseases which were at one time considered irremediable. I pledge myself to prove these assertions in the sequel, in opposition to every thing that may be urged to the contrary." 11.*

The opinions of medical men, even to the present moment, respecting the nature of puerperal fever, are various and dissimilar—some considering the disease as an inflammatory fever—some as a fever arising from extensive inflammation of some or the whole of the abdominal viscera—some consider

* Dr. Mackintosh considers himself authorized from experience to state "that four-fifths of the patients in a well-regulated hospital ought to be cured; and in private practice he feels convinced that at least two-thirds should be restored to their families."—P. 55.

Dr. Campbell and his pupils delivered, during the epidemic, 789 women, of whom 79 had puerperal fever, and out of these 22 died. Of the number who died there were only eight in whom bleeding and free purgation had a fair chance. In the other 12 cases the patients were too late in applying, or refused to submit to proper measures.

it as a putrid fever—others as a fever of the typhoid type, complicated with inflammation—while a fourth party look upon it as an affection *sui generis*, and peculiar to child-bed.*

*Symptomatology*. There is a very great uniformity not only in the symptoms but in the dissections of puerperal patients, as described by various authors; the discrepancies of opinion, therefore, respecting the nature and treatment, must have originated principally in theory and prejudice. There is good reason to believe, as Dr. Mackintosh observes, that puerperal fever is sometimes formed before the expulsion of the fœtus. We have seen more than two or three unequivocal instances of this ourselves—one of which is recorded in our quarterly series. Dr. Campbell has found that, in by far the majority of cases, "the disease appeared soon after parturition, generally within the third day." It very seldom came on so late as the 4th, 5th, or 6th day in Dr. C's practice. In all, except three cases, the disease was ushered in by rigors of various degree and duration—but generally so well marked as to be noticed by Dr. C's patients. On some occasions, however, it only amounted to a sense of chilliness or shivering. The shivering fit was soon succeeded by pain in the forehead and eye-balls, which became very distressing in Dr. Campbell's patients. Dr. Mackintosh, however, does not seem to consider this symptom as very uniform in puerperal fever, having witnessed several fatal cases where it was entirely absent.

Reaction now succeeds this cold stage, as in other fevers, with the usual phenomena of hot skin, quick pulse, thirst, and diminished secretions. A third stage generally shews itself more or less, with a perspiration on the skin, chiefly confined to the trunk of the body. But the great pathogno-

---

* We shall give the following classification from the very able work of Dr. Campbell:—

"By the following authors it is considered as an inflammation of the uterus: Hippocrates, Galen, Celsus, Ætius, Paulus, Avicenna, Raynalde, Felix Platerus, Sennertus, Riverius, Sylvius, Strother, Mauriceau, La Motte, Sydenham, Boerhaave, Van Swieten, Hoffman, Jussieu, Villars, Astruc, Pouteau, and Denman. By Hulme, Leake, and La Roche, as an inflammation of the omentum and intestines. Willis, Levrèt, Puzos, and Doublèt, consider the disease as of a peculiar nature. Peu, Tissot, Le Roi, and White, imagined the disease to be of a putrid nature. Petit, Selle, Kirkland, and Walsh, were of opinion that the disease was of a complicated nature. Finch, Stoll, and Doulcet, considered this affection of a biliary nature. Walter, Johnston, Forster, Cruickshanks, Bichat, Pinel, Gardien, Capuron, Gordon, Armstrong, and Hey, look upon it as inflammation of the peritoneum." 21.

monic symptom in this disease is pain, especially on pressure, in some part of the abdomen. This, however, is not, in general, complained of until after the appearance of the other symptoms; at other times, there is no distinct interval of ease between the *after-pains* and those which are *fixed*—the former degenerating, as it were, into the latter. Pressure with the hand should never be neglected by the practitioner; as the pain will otherwise sometimes escape the attention of the patient in describing her feelings. At the commencement of the disease the pain is pretty constant, but in cases advancing towards a fatal termination, intervals of ease have been remarked by almost all writers, and afford fallacious hopes to the patient, the friends, and sometimes to the inexperienced practitioner. The situation of the pain has been described as very various. In our own practice we have found it more frequently in one or other iliac region stretching across to the middle of the hypogastrium. This pretty nearly coincides with the experience of Dr. Campbell, who observes—

"In all my cases, there was pain in the hypogastrium at the commencement of the disease, darting into one or both iliac regions. In a few examples of this affection, patients described the pain as having commenced in one or other of the iliac regions, and extended towards the uterus, which organ felt enlarged, and was exceedingly sensible upon pressure. In my practice, therefore, I can with confidence assert, that the pain in the beginning of this affection was chiefly confined to the hypogastric and iliac regions. Patients never complained of it in the umbilicus or epigastrium except in one case,* until the disorder had continued for some time, and I am firmly of opinion, that those writers who describe the pain as having been chiefly seated in the epigastric region, in some instances, at the commencement of the disease, must have deceived themselves by confounding its stages. I trust, I may be excused for making this assertion, because from the number of cases I have treated, I must have witnessed the various modifications of this complaint." 32.

Dr. Mackintosh found the pain fixed in some part of the abdomen, usually the hypogastric region, or at one or other side, where the uterine tumour was generally recognized. The pain, from being sometimes slight at first, quickly became excruciating, so that the patient was unable to turn in bed, dreading the slightest touch, even of the bed-clothes.

"When," says Dr. Campbell, "we have not succeeded in arresting the progress of this formidable disorder, the pain gradually ad-

"* Vide Case II. of this Work, the only one where the patient complained of pain in the epigastric region from the commencement."

vances from the lower part of the abdomen to the umbilicus, and from that into the epigastric region, accompanied by short intervals of ease, but afterwards returning with increased violence, attacking the patient as it were by paroxysms such as I have already described. At the commencement of this disease, I generally found the abdomen more or less tumid, and this tumidity increased in proportion as the situation of the patient became more precarious, until the abdomen, in some instances, was as prominent as before delivery.* This happened in some of our first fatal cases, where the lancet had neither been so early nor so boldly employed as on subsequent occasions; but after we began to have recourse to bleeding earlier, and with greater freedom, the abdomen, although somewhat distended in every unsuccessful case, was not, however, enlarged to the same extent as the first. The uterus, in almost every instance, could be distinctly felt above the pubes, it was extremely sensible to the touch, and my impression is, that this organ increases in size during the disease; for on comparing in my own mind, the uteri of females in a healthy state, immediately after the placenta is thrown off, with those which I procured from the unfortunate victims of the puerperal fever, I am quite satisfied as to the correctness of the opinion I have now advanced."† 33.

From the beginning there is generally great derangement of the vascular system. In many of Dr. Campbell's patients the pulse continued frequent from the moment of delivery, and then the fever set in on the second or third day. The last-mentioned author considers the deviation from a natural state in the pulse to be the very first symptom in order of time, and that by carefully watching this, he has detected the disease, as it were, *in embryo*, and arrested its further progress without being obliged to have recourse to any very bold measures. Dr. C. has never found the pulse under 110 after the disease had been fairly formed—more frequently it was 120 to 130—and when the fever had continued some time it was seldom under 140. In the advanced stages of cases that terminated fatally the pulse was oftener above than under 140.

"In the commencement," says Dr. C. "the pulsation is sometimes full, but more generally hard; and as the disease advances, it becomes contracted or thready; frequently intermits; and towards the close, it is so weak for a considerable period as to be scarcely perceptible." 35.

---

* "The swelling of the abdomen having once begun increases very rapidly, insomuch that the belly will become as large as it had been before delivery."

† "The uterus was lying about the brim of the pelvis, and considerably more enlarged and distended than it ought to have been.—Gordon, Case III. See Cases XVII. XXVII. XXXIX. and XL. of this Work."

Dr. Mackintosh does not seem to regard the pulse as so good an index in this as in some other diseases. He invariably remarked, however, in the most acute cases, that the greater the ravages of inflammation in the abdomen, the sooner in the course of the disease had the pulse sunk and become feeble.

"Generally," says Dr. Mackintosh, "the pulse beats from 110 to 130 in a minute—sometimes full, at others weak and contracted. It frequently changes in the space of an hour from the former to the latter, when, unless energetic measures are quickly adopted, nothing can save the life of the patient. This circumstance, connected with another, has misled many eminent men in conducting the treatment of this disease, who, by reason of their other unavoidable engagements, cannot see the poor sufferer so often as is necessary." 40.

There is a peculiar expression of distress in the countenance of a puerperal fever patient which cannot easily be mistaken after it has once been witnessed. The eyes are destitute of animation—there is a degree of listlessness or indifference towards surrounding objects—the patient lies on her back, presenting an anxious desponding aspect. The face is occasionally flushed—the cheeks have a deep crimson appearance sometimes, while at others they are livid, and the patient seems exhausted—the eye, when the attack is severe, is frequently suffused with tears, and the pupil dilated.—*(Campbell.)*

The tongue, says Dr. Mackintosh, is generally moist and of a white colour, very slightly loaded. As the disease advances, it becomes dry and hard in the centre—in several cases he has seen it *very red.* Dr. Campbell describes the tongue as generally white and moist on the upper surface, except the raphe and margins, which, in severe cases, have a fiery red appearance—a state of tongue which he avers is highly characteristic of inflammation of the abdominal viscera.

In Dr. Mackintosh's practice the thirst was frequently urgent from the first, even where the tongue was moist. In some severe cases, however, thirst was not complained of. Dr. Campbell did not observe the thirst to be very troublesome at the beginning of the disease; but in the advanced stages of unfavourable cases it became very urgent, the patient being anxious for cold water.

The last-mentioned author observed the skin to be sometimes quite parched, and its temperature greatly increased when reaction took place—but these conditions soon gave way to partial sweats, the animal temperature not being much, upon the whole, beyond the natural standard, except after the first rigors had passed off. Dr. Mackintosh's observations on the state of the skin are to the same effect.

Respiration is always quickened in this disease, not, we should suppose, from any thoracic affection, but from the velocity of the circulation, the distention of the abdomen, and the pain which a full inspiration causes.

"The stomach and alimentary canal," says Dr. Campbell, "are organs which suffer greatly in this complaint. From the commencement of indisposition, there is nausea, rarely vomiting. According to my experience, the derangement of the stomach keeps pace with the abdominal pain. At first, therefore, there is only a degree of nausea, occasioned by the consent of the stomach with the uterus, but as the abdominal uneasiness increases in severity, and the pain begins to extend towards the upper parts of the abdomen, the nausea is converted into an actual vomiting, first of phlegm or frothy mucus, and ultimately dark coloured matter like the grounds of coffee. For some hours before dissolution, when the pain in the abdomen is excruciating, immense quantities of coffee-coloured matter are brought up almost without an effort."* 40.

The bowels are generally constipated, and continue obstinately so, until the disease is far advanced, when diarrhœa equally obstinate often succeeds. "In some of our fatal cases, says Dr. Campbell, I found it almost impossible to remove the torpor, and quite so to subdue the diarrhœa."

"The evacuations differ in appearance, I have observed them to be sometimes of a dark brown colour, at other times greyish or ashy, and very generally frothy; and whatever was discharged, had always a most intolerable odour. The diarrhœa is attended with severe griping, partly produced by flatus, but chiefly by increased and inordinate peristaltic action." *Campbell, p.* 41.

Dr. Mackintosh observed the stools to be generally dark-coloured, and extremely fetid. Sometimes they were yeasty, slimy, or watery.

---

* "Vomiting and sickness at the stomach are very usual symptoms, &c. what is thrown up, is of a green or blackish colour; when death approaches, there is continual vomiting of a green or blackish matter.—Hulme, p. 8. What the patient vomited was black, and had a strong resemblance to the grounds of coffee.—Gordon, p. 6. The rigor is often followed by nausea and vomiting of a bilious and sometimes coffee-coloured matter; in another place, in private practice, coffee-coloured matter is brought up almost without any effort.—MS. Professor Hamilton's Lectures. There was an almost perpetual vomiting throughout the second stage, though only a slight nausea occurred at the beginning, and very little vomiting in the rest of the first stage. Indeed, vomiting was always more urgent in the last than in the first stage of the disease, and the matter thrown up very much resembled coffee grounds, and was offensive to the smell.—Armstrong, p. 7."

The disease generally attacks before the afflux of milk to the breasts; and where this afflux has taken place, the mammæ become flaccid, and the lacteal, like all the other secretions, is diminished or entirely suppressed on the supervention of the fever. Dr. Mackintosh properly advises the early application of the child to the breast after parturition, in order to establish a determination to the mammary vessels, which may have some influence in checking irregular determinations to other organs.

The state of the lochial discharge has given rise to great controversies as to the reality or spuriousness of puerperal fever.

"Some say," observes Dr. Campbell, "that there is more or less of a suppression of the lochia in every example of the disease, others again, that it is altogether suppressed in some cases; while the distinguished Professor Hamilton declares that it does not suffer any change, and that the disease cannot be puerperal fever where the uterine discharge is suppressed.* My experience corresponds in some measure with this eminent individual, for in all my cases, except one, the uterine discharge was always present to some extent, and I have since been of opinion, that I had in this instance suffered myself to be deceived by the attendants, who often say that the lochia are suppressed when they really are not. Although this discharge continued to flow, to use the words of a celebrated author, there was always 'more or less of a suppression of it,' and this was particularly conspicuous immediately after the accession of rigors, a change naturally to be expected, as all the secretions are diminished during febrile excitement, not only in the puerperal state, but on every other occasion. I would not, however, wish it to be understood, that I should be so illiberal towards my brethren as to insinuate, that they have allowed themselves to be deceived in all their cases; for I should suppose that there are deviations to be remarked in the lochia as well as in every other symptom of the disease. It is an assertion extravagantly ridiculous for any person to make use of, that such judicious men as Denman, Leake, Gordon, Armstrong, and Hey, have confounded the puerperal fever with other affections, because they have stated the condition of the lochial discharge to be different to that described by one or two others." 44.

It certainly appears rather inconsistent when we find Dr. Hamilton recommending Hulme's cases to the world as being the most genuine and best marked specimens of real puerperal fever, while that very author expressly states that the

---

* "The lochial discharge continues, and in no case have I ever known it affected either in private or in public practice; and I am positive that when it was suppressed, the disease was not puerperal fever.—MS. Lectures of Professor Hamilton."

lochia are more or less diminished in quantity in the commencement of the disease.

Dr. Mackintosh found the lochia to flow, as in health, in a few cases; and in but a few cases were they entirely suppressed. Dr. Mackintosh here quotes passages from all the best writers on the disease, from which it appears that no positive conclusion can be drawn from the state of the lochial discharge in puerperal fever. All the authors, however, Professor Hamilton excepted, agree in describing the lochial discharge as more or less affected in the disease under consideration.

The urinary, like the other secretions, was generally affected, but not to any great extent in Dr. Campbell's practice. From the time the disease came to be well marked, the urine was diminished in quantity, and the patient complained more or less of pain in voiding it. Dr. Mackintosh makes nearly the same remarks, and the appearance of the urine was not very particular.

The blood generally exhibited a thick, firm, buffy coat in Dr. Campbell's cases. In the first blood drawn, the coagulum was large and tenacious; but in every succeeding detraction, it became smaller and smaller, still continuing firm, the quantity of serum increasing in proportion as the crassamentum diminished. "Mental aberration," says Dr. C. succeeded the free use of the lancet in four cases, but all of them ultimately recovered."

"One became deranged after losing forty-five ounces of blood at one bleeding, which subdued the disease, but she had been subject to puerperal mania after her former labours, and remained in a state of alienation on this occasion for nearly three months; a second, after losing eighty-six ounces of blood at three different bleedings, continued maniacal for fourteen days; another patient, after having been bled to sixty-six ounces, continued in a state of aberration for upwards of two months; and the fourth was delirious only for twelve hours, after having lost 128 ounces of blood." 46.

There was delirium in four cases only, three of which died. In two of these the delirium might be ascribed to improper treatment.

The period at which the disease proved fatal was very irregular. In neglected or improperly treated cases, it was astonishing how rapidly the disease ran through its different stages. Dr. Campbell heard of instances where the patients never rallied after the rigor, and sunk in 24 hours from the commencement of the attack. One of Dr. C's patients died in eighteen hours from the beginning of the rigor. This case affords a convincing proof of the reality of what has been termed and described as *congestive fever*, by Drs. Jackson, Armstrong, and others. We shall, therefore, extract the case and dissection.

"The patient alluded to, J. Gulry, was delivered of twins at five on the morning of the 21st of December, 1822, by one of my pupils, Mr. Patullo. At ten o'clock she was seized with a violent rigor, but we heard nothing of this circumstance, until the gentleman, by whom she had been attended during her labour, called on her the following day, and informed me soon after that he found her insensible and labouring under stertorous breathing, with scarcely any pulsation at the wrist. I visited her shortly after, she was then *in articulo mortis*, and died at four o'clock. The account I received from the attendants was, that 'she trembled from head to foot, that she never recovered her natural heat afterwards, that she refused every sort of nourishment, that she became delirious early in the morning, and that her breathing had become more or less oppressed from an early period after the time she shivered.' It may not be improper to state, that this woman was not married, and that she had two children formerly in a similar way. On the present occasion, her parents were so enraged at her conduct, that when her labour came on, they turned her out of doors about three in the morning during excessively piercing cold weather, and she was delivered in the house of another poor woman, without a single stitch except her body clothes to shelter her from the inclemency of the season. The body was examined by Mr. Lizars, in presence of Dr. Duncan, junior, Professor of Materia Medica, Dr. Mackintosh, and Dr. Orr. There was no effusion of coagulable lymph or serum into the abdominal cavity; nor was there any adhesion of the intestines or other viscera; and the inferior margin of the omentum, which, in almost all the other cases was charged with purulent matter, in this, however, appeared sound. The only thing remarkable, was great congestion of the intestinal and uterine veins. The spermatic veins especially, were so distended with blood, that they could be compared to nothing else than the ascending vena cava; and the veins ramified on the arches of the colon, were exceedingly turgid. The uterus was remarkably large, but contained nothing except portions of the decidua. This was the only instance I met with, in which reaction did not take place after rigors, but I had occasion to know that several cases of a similar character happened in the practice of other gentlemen in this city, during the late epidemic season. It may be doubted whether we are justified in considering the affection, of which I have now described a case, as a variety of the Puerperal Fever, or whether we should look upon it as a distinct disease." 49.

We look upon the above case, and all similar ones, to be nothing more than fatal instances of fever anterior to, or with imperfect re-action, of which Dr. Jackson first described numerous examples, and afterwards Dr. Armstrong in his valuable writings. Dr. Jackson's works have been so much overlooked by the profession, that we are here tempted to introduce two extracts from his writings—one, to shew the general features of congestive fever, and the other, to exhibit the usual appearances found after death.

(No. 1.) Phenomena of congestive fever.

"The symptoms," says Dr. Jackson, "commence with more or less of cold, the heat which succeeds is seldom high as judged by the hand, or as measured by the thermometer applied to the surface; the sensations of internal heat and internal burning are often insufferable. The skin is thick and torpid; the countenance is dark and grim, sometimes agitated, sometimes torpid and inanimate—bloated without expression—livid and of a peculiar gloss. The eye is usually clear, white, vacant, with an idiotic drunken stare; sometimes it is confused, agitated, and protruded. A sense of anguish at stomach, scarcely to be expressed in words, sometimes accompanied with nausea—sometimes without nausea, often distresses the patient in an extreme degree. Delirium occurs sometimes, and, when it does occur, it is furious, but the occurrence is not common. The pulse is sometimes irregular and irritated, impressing the idea that it is restrained from expansion by some latent cause of resistance; sometimes it is slow, sluggish, overwhelmed as it were by a load of oppression. The respiration is more or less disturbed; deep sighing is usual; gasping for breath, or an unceasing attempt to fill the lungs without the power to do it, is common; when present, it characterizes an aggravated form of disease. The tongue is often swollen, and, as such, incapable of distinct utterance; sometimes it is smooth, red, or rather livid; sometimes white and foul, the surface strewed with mealy patches; sometimes it is foul and leaden coloured.

"The above appearances are conspicuous under the tumult of invasion; and, under this tumult, convulsion sometimes supervenes, and the patient dies apoplectic before the close of the first day. But in general, the action assumes an ostensible febrile form; and, under that form, advances, with more or less regularity, until the third, and sometimes until the fifth day, when it usually terminates fatally."*

(No. 2.) Appearances on dissection.

"The following appearances were more or less observable in all, or in almost all those who died of this form of disease. The veins and sinuses within the head were turgid—distended with black blood; the choroid plexus appeared sometimes as an unorganized clot of blood; the lungs were frequently black resembling a sponge filled with blood—sometimes throughout, sometimes partially,—the substance was sometimes firm and dense, not unlike the substance of spleen. The veins of the omentum and of the external coat of the intestinal canal were distended throughout—the blood of a dark colour. The small intestines, as viewed exteriorly, often appeared black as if gangrened; the interior was filled with grumous blood—

* Dr. Jackson on Febrile Diseases, Vol. I. (2d Edition,) p. 91-2.

the coat of the intestine itself not diseased. The liver was often enlarged in size, distended with black blood, its substance rotten, its exterior coat sometimes ruptured by distention; the contents of the gall bladder were often of a pale colour and of a thin consistence; the spleen was generally large—the coats frequently ruptured—the interior a grumous mass." 89.

We have brought forward the above extracts in order to induce our brethren to consult more generally, the invaluable writings of the venerable and experienced Jackson. In them they will find an inexhaustible fund of accurate observations on febrile diseases, which the obscurity of the language in the earlier editions of his works prevented from being generally appreciated as they deserve.

Dr. Mackintosh occupies a considerable portion of his volume in tracing an analogy between puerperal fever and some forms of congestive disease under other circumstances. Thus, yellow fever, cholera, dysentery, &c. &c. are brought forward to illustrate his point, and, we think, he has not only stretched his analogies too far, but needlessly and improperly entered into the pathology, causes, treatment, &c. of diseases that had no relation at all, or extremely little, to the subject of puerperal fever. Thus, to give an instance of these strange and unnatural digressions, Dr. Mackintosh enters once more into the controversy with Dr. Chisholm; and the Hankey, the Bulam fever, and all the threadbare discussions about the contagion of yellow fever in the West Indies, are gone over again in a work on the Epidemic Puerperal Fever of Edinburgh. All this portion of the work we must pass over, and all this portion Dr. M. will do well to leave out in another edition, should the work arrive at that honour.

It is proper to state here, however, that Dr. Mackintosh insists upon "two great and striking varieties of this disease, which, by the symptoms of one running into those of the other, have tended more to blind the judgment of professional men, and lead them into false conclusions, than any other circumstance whatever." The first variety is the congestive form of the disease, which, however, is very rare. We shall give Dr. Mackintosh's description of it in his own words.

"In the first variety, there is a manifest oppression of the vital powers, commencing generally with a rigour, more or less severe—coldness of the surface, particularly of the extremities—paleness of the countenance, expression of anxiety in the features—a soft compressible pulse; which in some cases has a full beat; at others it is contracted—respiration sometimes laborious, at others amounting only to a slight difficulty. Pain in some part of the abdomen is complained of, for the most part over its whole surface, in some cases confined to the epigastric, or hypochondriac regions. In this state of the system it is sub-acute; increased, however, upon strong pressure—or, excessively acute, the patient dreading the slightest touch. The patient is quite sensible, although she speaks little; aware of her awful condition—she desponds. In some cases, diarrhœa precedes, or soon follows, these symptoms; and she dies, sometimes in six, sometimes in twenty-four hours, in spite of the exhibition of brandy, which it is so much the custom to rely on. This, it must be confessed, is a rare case; it does now and then however, occur, and after it has existed an hour or two, no treatment will prove effectual. Prevention here, is the grand point, and a good attentive practitioner can almost always see, where the disease is to be expected." 31.

These congestive forms, as we said before, of this and other diseases, are merely a more than usually depressed state of the sensorial power, and a languid state of the heart; in consequence of which, the re-action of the system, which in all cases, is a salutary effort of Nature, cannot take place, and the constitution sinks under the attempt. We consider this variety as entirely owing to a greater intensity of the cause of the fever, whatever that may be, as contagion, miasmata, malaria, &c. or a greater than usual debility or susceptibility of the constitution.

### PATHOLOGY OF PUERPERAL FEVER.

Considerable diversity of opinion has prevailed respecting the nature, or in other words, the pathology of puerperal fever. The ancients, without the aid of dissection, came to the conclusion that it was inflammatory—others, since their time, thought it to be of a peculiar nature, and proper only to women in child-bed. A third order of physicians, supposed it to be of a putrid nature—a fourth, bilious—and, lastly, another class consider it as the common infectious fever, complicated with a more or less extensive inflammation of the peritoneum. Dr. Campbell, in proceeding to analyze these opinions, grounds his arguments entirely on the symptoms, the dissections, and the effects of treatment, laying theory aside altogether. That the ancient opinion was the most correct one, will probably appear in the sequel.

"With respect to the first opinion," says Dr. Campbell, "every symptom and feeling of a patient, from the moment the disease can be said to be ushered in, clearly show that it is of a highly inflammatory character. If rigors, quick firm corded pulse, acute fixed pain, tumefaction, and increased heat, be considered as constituting the leading symptoms of the definition of inflammation, we cannot surely deny that the disease under consideration is of this nature, for almost all of these symptoms are present in a prominent degree throughout all its stages. When we are afforded opportunities of examining the bodies of those who fall victims to the disease, we then have, in consequence of the great devastation of many, or on some occasions, of all the parts contained in the abdominal cavity, the most undeniable proofs of extensive inflammation. In some cases the vascularity of all the parts is increased, although not to such extent as to account for the fatal event. In other instances, the evidences of increased vascularity are only remarkable at particular points, the change of structure being confined to the uterus, or to its appendages only, or to the intestines, omentum, and peritoneum. But even in those cases, where the appearances of excitement were by no means considerable in some parts, other organs, however, such as the omentum, intestines, and ovaria, in the same case, were in a state of suppuration, or approaching to gangrene. Independent of the very obvious marks of excitement which I have now particularized, we meet with others not less characteristic of the inflammatory action, viz. the effusion of coagulable lymph and serum.

"It will be seen by the dissections which I have detailed, that the effusion of serum and coagulable lymph was very considerable, exceeding on some occasions several pounds in quantity; appearances which have been noticed by every person who had opportunities of examining the bodies of those who fell victims to this affection, and particularly by the late very accurate Dr. John Clarke, who, in consequence of the great quantity of matter thrown out, was disposed to attribute the effusion, not to active inflammation, but to a peculiar action of the peritoneal vessels, quite distinct from this morbid state. Effusion, however, is now universally looked upon as one of the terminations of inflammation, and whoever will take a correct view of matters, in reference to the previous state of the subject before effusion can be said to have taken place, such as the repeated accession of rigors, the acceleration of the pulse, the acute fixed pain, and ultimately the progressive enlargement of the abdomen, must be convinced, laying aside all other proofs, that nothing but violent excitement could produce so great an accumulation of serous fluid. Will any one of the present day attempt to deny that effusions into the brain and thorax, are not the effects of previous inflammation, and in either of these affections are symptoms of excitement more distinctly marked, than in the disease under consideration? And as to the preternatural membrane, or crust which covers the abdominal viscera, do we not often observe similar appearances on the surface of the lungs, of the heart, and of the liver, in cases where those organs have been the seat of inflammation?" 174.

In some instances, Dr. Campbell observes, the excitement proceeded so far that the abdominal viscera had contracted adhesions with one another by abundance of coagulable lymph deposited between their convolutions and into their different interstices. To the extensive effusions of serum and coagulable lymph may be ascribed the little apparent increase of vascularity, and the want of turgescence of vessels; "for in cases where those extravasations were limited, the characters of inflammation, such as engorgement of the vessels and change of structure from actual suppuration, were more distinctly marked."—*Campbell.*

"In every one of our dissections," says Dr. Campbell, "the evidences of inflammation were so decisive, that I have no hesitation in declaring, were I to invite a mere tyro in physic, after having heard lectures on the ravages committed by inflammation, to witness the examination of one of these bodies, and to ask him what did such a patient die of, he would at once reply, inflammation." 175.

Dr. Mackintosh, after reviewing the various pathological doctrines which have been broached by former writers, states that the pathology which he himself has been led to adopt, is derived from an analysis of the symptoms, and the undisputed fact that puerperal fever is less dangerous the later the attack after delivery—and that it is still milder after abortion. His opinions are also strengthened by the analogies which the fever bears to other inflammatory and congestive diseases.

"I agree," says Dr. Mackintosh, "with the sentiment so well expressed by Gardien, that, in the consideration of this disease, *it is impossible to separate its history from the condition of the female after parturition.* That it is not a disease *sui generis*, I think, is sufficiently proved by the facts hitherto detailed; and it appears equally evident from these, that the disease is nothing more than an inflammatory affection of the peritoneum lining the cavity of the abdomen, and which covers its contents; commencing, it is most probable, in some portion of that membrane which covers the uterus, tubes, and ovaria, accompanied with a greater or less degree of congestion of the blood, which is sometimes so great, as to kill the patient before reaction takes place. This tendency to congestion is greatly increased by the state of a woman immediately, or soon after delivery. It is simply this state that renders, and, that ever will render this disease so formidable, in all quarters of the globe—in Greece, as well as in other parts of the continent—in Britain, as well as in its foreign possessions—in South and North America. In many of these situations, except the first and the last, I have watched the progress of, and examined the changes of structure produced by, this disease, in the bodies of those who fell victims to it. I can aver, that I have seen above one hundred cases, and about thirty dissections; and I am enabled to state, that although the symptoms do vary in degree and duration, the appearances on dissection are almost always perfectly similar. I do not, by this, mean to impress a belief on the mind of the reader, that this or that part of the peritoneum is more diseased than another. All that I wish to convey, is, that the appearances do not vary more than in peritoneal inflammation, in men, or in women in the non-puerperal state. It is, therefore, this peculiar state of a woman's system, after child-birth, that is now to be investigated." 169.

Dr. Mackintosh makes many judicious remarks, physiological and pathological, on the puerperal state and the probability of its predisposing to, and aggravating peritoneal inflammation. He agrees not in the fashionable pathology which attributes every disease to certain changes or commotions in the vascular system, to the exclusion of participation in the nervous system. Our readers are aware how strenuously we have, at all times, opposed this confined view of febrile pathology; and not only opposed it, but endeavoured to show that in almost all fevers the nervous system was the first to receive the shock, and its functions the first in order of derangement. We extract the following passage from Dr. Mackintosh as containing much sound reasoning.

"In considering the state of the vascular system in a recently delivered woman, we must have recourse to the changes which take place with conception, and which continue to go on during gravidity till the full time, before we can understand what takes place afterwards. I consider it as an established fact, that, from the moment, or soon after conception takes place, an increased action of the vascular system comes on, and that a necessary determination of blood to the uterine region commences. Menstruation is suspended, which increases the general tendency to plethora. In the early months, we find nature struggling to counteract, to a certain extent, this new determination, and this recently-acquired plethora, by a constant nausea and sickness, which not only diverts the blood to the surface of the body, but also prevents an increase in the quantity of that fluid, little food being allowed to remain on the stomach. The physician often finds it necessary to give his assistance to one side or other; which, unless he does timeously and judiciously, the balance of the circulation will be entirely upset, and the only way by which nature can relieve herself, is either by diarrhœa, or a spontaneous discharge of blood from some part of the body, or by miscarriage. This general plethora exists, more or less, up to the full time, and the determination to the uterine region goes on gradually increasing to the same period; partly, to supply the means of growth to the uterus and its appendages, and partly, to support the change, which it is believed, takes place on the blood of the fœtus in the placenta. In consequence of this, the quantity of blood transmitted through the abdominal aorta, becomes very great: the blood-vessels of the viscera receive more than the usual quantity; but this is prevented to a considerable degree, by the equal pressure afforded to them, as the circulation increases, by the increasing size of the womb itself.

"The uterus is supplied with blood by the spermatics and hypogastrics, which greatly increase in size.* The veins accompanying

---

* "'The arteries, both hypogastrics and spermatics, are very much enlarged.' *Anatom. Description of the Human Gravid Uterus*, by William Hunter, M.D. p. 16."

the course of the arteries, have the same name, and are still more enlarged in size than the arteries; their immense size has induced many anatomists to call them sinuses. These, as well as the veins in the cavity of the abdomen, are without valves; they are more distended in the gravid, than in the ordinary state of the system; and, I apprehend, that the blood will find increasing difficulty in returning to the heart from the uterine region. This will explain many of the anomalous symptoms which occur during gestation, and the relief generally afforded by blood-letting; and will bear me out in the assertion, that the calibre of these vessels is increased, to perhaps double their natural size.

"There is also a peculiar change in the nervous system during utero-gestation, which shews itself so early as the first weeks of pregnancy, and goes on augmenting till the period of its termination. This has been very aptly styled, the increased susceptibility of impression of the nervous system. This morbid change, if I may say so, chiefly displays itself in the temper; and, by slighter causes, producing more violent irritation, both of mind and body, than in the unimpregnated state." 173.

During labour a febrile state, to a greater or less extent, is produced—and afterwards blood is lost, varying from twelve to forty ounces, or more, which, in all probability, is a salutary evacuation when within moderate bounds. The abdominal and uterine plethora is now also relieved by a tide of the circulation and excitement to the mammary vessels. During all these curious changes and revolutions the nervous system is in a state of great irritability or susceptibility. The least noise causes great agitation, and disturbance in the circulation, and it is but too well known that frights, despondency, and other mental impressions have, in all ages, produced the most fatal consequences in the puerperal state.

"And when we consider the plethoric stase af the system of a woman with child—the determination of blood so long continued to the abdominal region, which is suddenly checked after the birth of the child—the susceptibility of the nervous system to receive external impressions—and the sudden removal of the pressure which the large uterus produced on all the parts within the abdomen,—we shall, at a single glance, perceive how causes, such as the application of cold, too early sitting up, irregularity in diet, imprudent use of stimuli, or anxiety, grief, and horror, or even very slight affections of the mind, in an irritable system, in which the balance of the circulation is not yet confirmed, may upset it altogether, by causing an irregular determination of blood, which will very naturally be directed to the vessels most ready to receive it." 179.*

We are ready to grant that these and other explanations

---

* Dr. Mackintosh, p. 178-9.

are applicable enough to puerperal fever in its sporadic form; but Dr. Mackintosh makes no attempt to account for the epidemic form of the disease, and consequently leaves out the main point of the etiology. Dr. M. no doubt saw that the moral and physical circumstances above alluded to, could not possibly differ so much in one year from another, around the women of Edinburgh, as to make all the difference between health and wide wasting disease. He has therefore passed over the subject of epidemic influence, leaving it unexplained—and what is worse, leaving unexplained the modifying influence which this epidemic constitution operates on the nature and treatment of the disease. This is the great failing of ardent young practitioners. They cannot bear to think that diseases vary with the season and with the epidemic character—a truth which the more experienced and more sober part of the profession are convinced of from daily and yearly observation. On this account, and in this respect, Dr. Mackintosh's etiology, and even his practice, is defective; for we are old fashioned enough to believe, nay to know, that there may be *inflammations* which, under certain epidemic constitutions, can be more readily cured by bark than by the lancet. We must not, therefore, lay down a sweeping pathology and therapeia that will admit of no modification on account of times, places, and circumstances. In throwing in this caveat we do not mean to say that the late epidemic was one in which bleeding was unnecessary—far from it. But we mean to say, that every practitioner, at the commencement of an epidemic of any kind, should act with caution, like Sydenham of old, till he ascertains the character of the disease he has to treat—and not go to it with a firm persuasion that it can have but one character, and consequently but one mode of treatment. Indeed, the various, and sometimes diametrically opposite remedial agents which every old practitioner must, in his time, have seen prescribed for, and curing fever, cannot but leave a conviction on the mind that diseases assume an almost infinitely varied character, from time to time, and require corresponding modifications of treatment—and that even when their pathology appears uniform.

Dr. Mackintosh, in his chapter on pathology, gives a sketch of the opinions of preceding writers on this subject, and then relates several cases from the practice of himself and others, with the appearances on dissectson, tending to prove the inflammatory nature of the disease, and the necessity of vigorous depletion in its treatment. For these cases we must refer to the work itself.

*Seat of the Disease.* Dr. Campbell states, that whoever will attempt to fix the seat of inflammation in puerperal fever, by dissection, will find himself disappointed—for, if we suppose the part most diseased is that first affected (which is a natural conclusion) we shall have the primary seat of this complaint in a different part in every three or four cases. In one case the uterus, in a second its appendages, in a third the intestines, in a fourth the peritoneum, and in a fifth the omentum will bear the principal marks of the disease.

We agree with this able author that it is more a matter of curiosity than practical utility to ascertain the primary, or even the principal seat of the phlogosis. If we are convinced that inflammation actually exists, the same means will cure it (if it can be cured) whether it be seated in one or other of the abovementioned parts. Were Dr. Campbell to give an opinion on this point, grounded on the symptoms and dissections, he would say that the inflammation *generally* begins in the uterus. The very circumstance of pain in the hypogastric region or ilia being among the first spmptoms, together with the diminution of the lochial discharge, appear to him to be sufficient proofs in confirmation of the above opinion. Whether the inflammation commences in the substance or in the peritoneal covering of the uterus it is difficult to say. Dr. C. thinks it sometimes begins in the one and sometimes in the other. In every case where our author was early called in, pain was complained of in the region of the the uterus, and this organ enlarged could be distinctly felt through the abdominal parietes, hard, and exquisitely tender on pressure. In many cases the pains seemed to radiate from the uterus to other points; and it was not until after the disease had existed for some time, that the uneasy sensation became general and stationary all over the abdomen. Dissection generally corroborated this idea—not in all cases, from the vascularity or change of structure in the uterus, but from its generally enlarged size.

Dr. Campbell makes some reflections on the name which should be applied to the disease under consideration, in order that no false impression might be conveyed with the term. He decidedly objects to "puerperal fever," to "low child-bed fever," and some other appellations calculated to mislead the young practitioner. "Peritonitis," he thinks, is preferable to all other terms hitherto applied.

*Predisposing Causes.* These have been touched upon already in the extracts which we have given from Dr. Mackintosh's work. Of the increase of the abdominal and uterine nerves, from pregnancy, Dr. Campbell had numerous opportunities of proving by dissection. Of the increased quantum of albumen in the blood of the pregnant female, there can be no reasonable doubt entertained. Dr. C's other observations on the predisposing causes, are very nearly the same as those of Dr. Mackintosh, to which we have already paid attention. We may state, however, that Dr. Campbell only observed two cases of puerperal fever where uterine hæmorrhage had taken place.

*Exciting Causes.* These, no doubt, are numerous and diversified, as in the case of any other inflammatory affection.

"Among the exciting causes," says Dr. Campbell, "which have been usually remarked as possessing influence in producing the disease in question, may be mentioned, applying the binder round the abdomen with unusual firmness after parturition, the sudden and forcible detachment of the placentary mass, and retention of the secundines, injuries before and during the process of parturition by brutal violence, or the use of instruments, and consequently severe labour; mental emotions, exposure to cold, premature use of stimuli, whether food, or drink, impure diet, lactiform metastasis, infection, and a noxious constitution of the atmosphere. Of the three latter, it will be necessary to take particular notice, as they have been supposed by different persons to have a considerable share in producing Puerperal Fever; but with regard to the others, it will suffice merely to offer some general remarks, as their power in exciting inflammation is universally acknowledged, and because I think I shall be able to adduce satisfactory proofs that some of them have had much influence in producing the present epidemic, or aggravating its symptoms after it did appear." 202.

The binder, if carried to an ultra extent so as painfully to compress the abdominal viscera, might be injurious; but, moderately and properly applied, it cannot be otherwise than very useful. In respect to the placenta, although no violence is justifiable, our author agrees with Dr. Hamilton, that it ought never to be allowed to remain more than an hour. It appears from our author's experience, that women who had been delivered for the first time, and whose labours were *generally* more severe than on subsequent occasions, suffered considerably more from puerperal fever, than those who had several children, and who must have experienced easier labours. The first patient, (Mrs. Hepburn) Dr. Campbell had every reason to believe, became affected with the fever in consequence of the severity of her sufferings during parturition.

On mental emotions we have already remarked. Puerperal patients are unquestionably more susceptible of impressions from cold than other patients. Many instances of this occurred in Dr. Campbell's practice. Some instances, also, were remarked, where the inordinate use of stimuli brought on the disease.

On the subject of infection our author feels great reluctance in expressing his sentiments, as the conclusions he has drawn are in direct opposition to the opinions of men of the first rank and eminence in their profession. From what fell under his own observation, Dr. Campbell does not feel himself authorized to concur with the contagionists. Only about one in ten of his patients were affected with the disease;—and, as neither he nor his assistants used any particular precautions to prevent the dissemination of contagion, if such existed, he thinks this fact goes strongly against contagion. The following communication, which Dr. Campbell received from Mr. Syme, a practitioner of eminence in Stirlingshire, appears to us to be somewhat indicative of the occasional infection of puerperal fever.

"When this chapter was printing, I received from Mr. James Syme, a practitioner of eminence at Alva, Stirlingshire, the particulars of four cases of Puerperal Fever, drawn out with great accuracy. Two of which occurred in his own practice, the other two in that of Mr. Galloway, a surgeon of respectability in the same place. The symptoms were precisely similar to those observed in my patients. One died on the second, two on the third, and one on the fifth day from the commencement of rigors. Two were delirious for some time before death; and in the other two, vibices were observable on various parts of the body, as remarked in Case V. of this work. The lochia were greatly diminished in the progress of the disease, but not suppressed. The most remarkable circumstance, however, attending one of these cases is, that the child, a male, died in a few days after its mother, and that a woman who waited upon them both, and afterwards assisted in cleaning the bed-clothes, was seized at the end of three days with shiverings and other symptoms, similar to those observed in the case of the female she attended, and she likewise fell a victim to them in forty-eight hours. Another woman who had been hired about a week after the decease of this last, to assist in washing blankets, was also attacked with symptoms resembling Puerperal Fever, and in a few days shared the same fate with the other, although neither were pregnant nor nursing at the time. A similar thing happened in the Lying-in Ward of the Royal Infirmary here, during the time of Professor Young, and likewise at the Hotel Dieu of Paris. At the latter place, however, they were chiefly men who were affected. It may be thought by those who suppose this disorder to be infectious, that what I have just related is a strong corroboration of this doctrine; but in the present day, it is not necessary to adduce any proofs to show that inflammatory diseases often prevail epidemically, without being contagious." 227.

Without having seen direct and unequivocal evidence of the contagion of puerperal fever ourselves, we confess, that we have little doubt that it occasionally puts on that character. We have seen erysipelas over and over again decidedly contagious; and, if so, why should not puerperal peritonitis be sometimes contagious? We believe that it is only under peculiar circumstances, the nature of which we are yet ignorant of, that either of the diseases just mentioned, take on this dangerous character; but this is also the case in several other diseases.

Dr. Campbell very properly attributes the spread of puerperal fever, on such occasions as that lately witnessed in Edinburgh, to some epidemic influence, the nature of which is yet a secret.

"The ravages of this epidemic," says Dr. Campbell, "were not confined to the human species alone, but extended their influence even to the lower animals. Bitches in several families in town, soon after having brought forth their young, refused to suckle them, and died in two or three days after. A similar circumstance occurred in London, during the epidemic described by Dr. John Clarke. This, however, is an occurrence which may happen independent of any epidemical constitution of the air. In the winter of 1820, when the menagerie of Mr. Wombwell was in this city, several of his collection died a few days after having given birth to their young. A lioness in particular, on the second day after bearing two cubs, shivered, was taken very ill afterwards, and not only refused to suckle them, but actually pushed them away from her, and she died on the third day after having been seized, apparently in great suffering. There was no epidemic of any description in Edinburgh during that winter, and the animal's disease was ascribed by the keeper to her having caught cold. The uterus and broad ligaments, which I have now in my possession, were very much inflamed; and the peritoneum was affected in a similar manner. In various parts of the country, more especially in Fifeshire, the mortality among cows after calving, was remarkably great about the same period that Puerperal Fever was prevalent; for several farmers lost five or six cows, which was considered as a very unusual proportion. These are strong coincidences, and, in my opinion, go a great way to prove that the disease was produced by some peculiarity in the constitution of the air, and not by infection, or any other cause operating solely." 229.

*Diagnosis.* We entirely coincide with Dr. Campbell in condemning the pragmatical dictum of Professor Hamilton, who insists that the lochial discharge is always uninterrupted in real puerperal fever. We should not like to say any thing harsh upon this occasion; but we cannot suppose, that a man of Dr. Hamilton's penetration, on other occasions, can be absolutely convinced in his own mind, of what almost universal experience gives the negative to. We can only account for this obstinacy by the well-known *tenax propositi* which, in some individuals, is not to be eradicated from the mind by any possible means. We would beg leave to remind the Professor, that his pupils, when they go abroad in the world, have not his reputation to screen them when they commit themselves at the bed-side of sickness; and we can assure him that it is not many months since the character of one of them, and probably his prospects through life, were left at our mercy by a blunder, involving the life of a fellow creature, and based on this destructive principle of diagnosis, inculcated by Dr. Hamilton. The lesson taught this gentleman by the astounding appearances on dissection, will not easily be obliterated from his memory, and we warn the rising generation against the fatal delusion into which they are led, by mortified pride and unpardonable pertinacity.* It is most true, as Dr. Campbell properly remarks, that the symptoms of the disease teach us, and the dissections confirm, that distinctions into low child-bed fevers, peritonitis, hysteritis, and enteritis, are of no practical utility, because all these parts are so intimately connected that, wherever the inflammation begins, the excitement cannot long be confined to any one part, but must spread with rapidity over the whole.

"To the younger part of the profession," says Dr. Campbell, "I would offer a diagnosis for this disease, which for simplicity they cannot mistake, and for accuracy will stand the test of experience, by which alone we should be guided. When a practitioner, therefore, meets with a puerperal patient labouring under *acute fixed pain in the lower part of the abdomen, aggravated on pressure, or a general soreness of the abdomen rendered more acute by pressure, accompanied with frequent pulse, hurried inspiration, and much uneasiness on turning to either side in bed, he may rest assured that such patient is affected with Puerperal Fever;* and unless she is considered in this light, the conduct of the practitioner should undoubtedly be brought under the cognizance of legal investigation for professional ignorance, since *the symptoms which I have now enumerated must always be present in some degree.*" 237.

No condition of the circulation *solely*, ought to be relied on as a diagnostic, for the state of the pulse is as variable as any other individual symptom. Neither can the state of the stools be much depended on. They are sometimes, though not very often, frothy and yeasty—more frequently dark and offensive.

The only distinctions which Dr. Campbell insists upon in this disease, are sporadic and epidemic, in which he would

---

* "Why he should maintain that they (the cases detailed in the late epidemic) are not cases of puerperal fever, I cannot understand, unless, perhaps, with a view to account for his own acknowledged want of success in this disease."—*Campbell, p.* 232.

iuclude inflammation of the abdominal lining, uterus, and intestines.

"But I repeat, that any distinction of this nature ought not to influence the conduct of the practitioner, for the treatment of both must be the same; with this difference, however, that in the epidemic, it may be found necessary to carry our remedies to a greater extent." 241.

Here Dr. Campbell details a case which happened in Professor Hamilton's own practice, and was pronounced to be puerperal fever, where bleeding was objected to, where the patient died, and where dissection shewed the peritoneum injected, the mesenteric arteries exceedingly turgid, portions of the ileum inflamed—the mouth and neck of the vagina greatly so—the right ovarium containing a sac of purulent matter, and partly gangrenous, &c. He then compares this case with one from Dr. Gordon, proving their complete similarity, though Dr. Hamilton denies that Dr. Gordon's cases were those of puerperal fever. He also invites the reader to compare the case in question, with those related by Drs. Denman, Leake, Armstrong, and Mr. Hey, convinced that no man of impartiality will say, these distinguished physicians were mistaken in calling the disease they describe puerperal fever.

*Prognosis.* In all diseases, but especially in puerperal fever, our prognoses ought to be guarded. They ought to be like the oracular responses of old—capable of bending so as to quadrate with the event, whatever that may be. There is great art in doing this. If you are too mysterious, you may get the name of giving, or of *having* no opinion at all. If you are too clear and decided, you will unquestionably be so often wrong, (however clever) that your pride will be mortified, and, probably, your judgment impugned by those who know not the difficulty of predicting safely in human diseases.

While Dr. Campbell acknowledges puerperal fever, to be one of the most fatal, he still asserts that, if taken in time, and treated properly, it is "as curable as others which were at one time considered irremediable." We hardly understand this round-about comparison. That our prognosis will depend much on the time we first see the patient, is sufficiently evident. Dr. Campbell never succeeded, except twice, in saving any patient who had been upwards of twelve hours under the influence of the disease, before medical treatment was commenced. They saved few, indeed, who had been so long as six or seven hours ill. An early attack is dangerous, as was before observed. Long continued rigors denote a formidable disease—and so does a repitition of them. Great uneasiness and tumefaction of the abdomen—pain advancing towards the umbilicus and epigastrium with laborious respiration—obstinate constipation—early diarrhœa with tumefaction of the abdomen—black vomit—great loquacity—a hurried incoherent manner of speaking—delirium—total indifference to surrounding objects—inability to turn on either side—brown dry tongue, with sharpness of the features, are all looked upon, and justly so, by Dr. Campbell, as denoting danger. Some of them indeed, as the black vomit, may be regarded as fatal.

When we see the patient within an hour or two, says Dr. Campbell, after the accession of the rigors, we may make a favourable prognosis, provided we act upon principle, and without timidity. "An attack from the end of the third day from parturition is seldom fatal." Only two patients out of the whole number were lost under the above circumstances. We may also, Dr. Campbell avers, deliver a favourable prognosis when puerperal fever supervenes upon uterine hæmorrhage unconnected with external violence. Puerperal peritonitis succeeding abortion or premature labour is a basis for favourable prognosis.

"An early diarrhœa," says Dr. Campbell, "is always to be considered a good omen, provided the individual has been bled as freely after this state of the bowels has appeared, as if no such symptom had been present. Others again only look upon this symptom as favourable early in the disease, when the pulse diminishes in frequency, and when the abdominal pain and tumefaction subside. The disorder is always modified by an early purging; and I met with several cases to convince me, that one or two smart bleedings will subdue it entirely in such examples; but I have detailed others which clearly prove, that an early diarrhœa will not save the patient without the use of other remedies." 257.

Ability to turn in bed without assistance—a clean moist tongue, with general perspiration—return of the milk to the mammæ, and of the lochia, when either have been suppressed—the pulse continuing below 100 after bleeding, these are all favourable omens.

### METHOD OF TREATMENT.

Dr. Campbell remarks that when we consider the symptoms of puerperal fever, and the almost uniform proofs of inflammation which dissection discloses, it is astonishing that blood-letting should still have opponents. Both Dr. Campbell and Dr. Mackintosh imagine that much of this opposition results from a hypothetical and erroneous supposition that puerperal females cannot bear venesectionary depletion so well as under other circumstances. But without wasting time in these inquiries we shall proceed to lay before our readers the methodus medendi of the authors under review.

" Should," says Dr. Campbell, " a practitioner be on the spot during the rigors, I conceive that immediate steps should be taken to diminish the cold fit, by exhibiting *mild* warm diluents, such as weak tea and barley-water, with a view to determine towards the surface, and equalize the circulation. Besides the exhibition of warm diluents, we should recommend bottles containing hot water, to be placed round the patient; also hot bricks, in order, as much as possible, to obviate the effects of the rigor; and we afterwards proceed as circumstances shall direct. We are rarely so fortunate, however, as to be present at the accession of rigors; for I have always experienced the utmost difficulty in convincing patients of the necessity of acquainting the practitioner with such an occurrence.

" If ever emetics have proved beneficial, it must have been during the cold stage, by diminishing its violence, and restoring the action of the superficial vessels; but it must be allowed, that they are harsh remedies at so early a period after parturition, as that at which this disorder often shews itself. Among the better ranks, or in the lower sphere of life, when it can be procured, I am certain that the warm-bath will be found extremely useful during rigors; or a blanket wrung out of hot water should be tried. The latter will, perhaps, be preferable, for the exertion of removing the patient from bed, and placing her in the warm-bath, would tend to aggravate the disease, not to speak of the probable bad effects of exposure to cold.

" When we are called after the patient has shivered, we must be guided entirely by the state of the circulation. When the pulse is *firm and regular*, we should not hesitate to use the lancet, at whatever time we are applied to; for, if the individual *must* sink under the disease, nothing surely can be worse than death. I have detailed a case where the woman recovered, although she had not been visited for twenty hours after the disorder began; but, in other instances we were unsuccessful, although they had been attended to in less than six hours from the commencement of indisposition. In every example where we are called within a period of six hours from the accession of rigors, or of the manifestation of abdominal pain, we should bleed the patient immediately to syncope, an effect which was not readily produced in our cases. In the whole, there were only three or four examples of persons having fainted from a detraction of less than twenty ounces of blood, and there were many among them far from being stout or plethoric." 263.

When we are applied to at this early period of the disease, Dr. C. remarks, we ought not to bind up the arm until we have effected syncope, or, at all events, until the pulse is materially affected—and if it requires only ten or twelve ounces to produce this effect, venesection ought to be repeated whenever the patient recovers from her state of prostration, and fainting should again be brought on.

" In any case," says Dr. Campbell, " where a patient has been longer indisposed than what I have specified, the condition of the circulation alone is to be our guide during the flow of blood, and the arm must be secured whenever the pulse begins to flutter. I have often heard men of great experience say, and I have had many opportunities of knowing their sentiments to be correct, that we seldom do harm by bleeding too much, but very frequently by bleeding too little. I wish this to be particularly kept in view in the disease under consideration; for I am persuaded, that, if our assistance is early called for, we have very little to apprehend from using the lancet too freely; and I am no less satisfied, that in some cases, the contracted or apparently enfeebled state of the pulse, so characteristic of abdominal inflammation, has often deterred persons from performing venesection when it might have been done with advantage." 265.

Some timid practitioners have urged the plea that too copious abstraction of blood will produce effusion, and that thus we may be deceived in our post mortem researches. Whoever has paid proper attention to this subject must know that the effusion from extreme bleeding is of a very different kind from that produced by the excitement or inflammation of a membrane. We have seen some cases certainly where effusion was produced, in all probability, by the intemperate use of the lancet—but it was a clear lymphous effusion in all the cavities—in the chest, abdomen, and cerebral ventricles, without any marks of excitement in these parts. The inflammatory effusion, on the other hand, is always more or less turbid—very generally containing flakes of coagulable lymph, or apparently dissolved purulent matter. There will also, in most cases, be marks of excitement in the serous membrane whence the exudation issued.

The quantum of blood to be drawn, and the repetitions of venesection must depend on the urgency of the symptoms and the state of the circulation. Dr. Campbell observes that he never had cause to repent of blood-letting, even when employed at such a late period as to be without hope of success. Under these circumstances it acted as an euthanasian measure, mitigating the sufferings of the patient, though probably accelerating a little the final issue of the case.

"When the abdomen," says Dr. Campbell, "is *much relieved* by the first detraction, when the patient can cough and breathe with more freedom, and when she is able to turn with greater ease in bed, these are sure demonstrations that the first bleeding has succeeded to a very considerable extent in subduing the disease; and, under such circumstances, venesection need not be repeated while this state of matters continues, but the patient ought to be carefully watched, and the abdomen examined by the practitioner at every visit." 266.

When called in time, one smart bleeding has often, with proper auxiliaries, subdued the disorder, as may be seen in eight or nine of the cases detailed by this author. But should the abdominal uneasiness and other bad symptoms continue after the first bleeding, venesection must be repeated at the end of three or four hours at most, and carried a second time to the extent of syncope. The practitioner is cautioned by Dr. Campbell against being misled by any trifling alleviation of symptoms. As long as there is the least sensation of pain in the abdomen no patient can be considered safe. Even a third or fourth bleeding must take place, if the desired relief be not obtained by the first or second. In general it happens that at each succeeding detraction of blood after the first, the loss of a very few ounces will suffice to subdue the activity of the vascular system; and it is only in the severer cases, that it will be necessary to bleed oftener than two or three times. Dr. Campbell properly remarks that it is the state of the abdomen which must determine the necesity for bleeding, and the pulse, the measure or propriety of it. We need not always expect to diminish the *frequency* of the pulse by bleeding; for in a great many instances it is accelerated by venesection, and continues so for some time after the patient is convalescent. It may be repeated, that the bleedings must be reiterated "so long as the abdomen indicates such treatment to be necessary, and so long as the strength of the pulse will support it."—*Campbell.*

Dr. Mackintosh's directions respecting this paramount measure in the treatment of puerperal fever are almost exactly the same as those of his brother practitioner Dr. Campbell. Dr. M. insists on the necessity of seeing a patient under this disease every two hours at the commencement of the illness, otherwise no treatment, he thinks, will do any good. This attention, however, cannot reasonably be expected in the time of an epidemic, where the practitioner has other patients and other diseases to take care of. Dr. Mackintosh cannot be acquainted with the harrassing exigencies of *private* practice, or he would not lay down so rigid a rule for his brethren. At the same time we advise the practitioner, for his own sake as well as that of the patient, never to remain four hours without visiting a patient in puerperal fever, during the first and second day.

"It must be impressed upon his mind," says Dr. Mackintosh, "that nothing will be of use, unless bleeding is practised within the first six hours, and repeated every two or three, in such quantity as the nature of the disease requires, and the strength of the constitution will admit. It will never do for a physician to walk in, and order the precise quantity of *sixteen ounces* to be abstracted, and walk out again, leaving the patient in the hands of a person, whose experience, perhaps, is too limited to enable him to judge for himself, or whose youthful appearance is not calculated to enforce respect.—When I find it necessary to abstract blood, some impression is always allowed to be made on the system, or on the disease, before the arm is tied up. If syncope takes place in an early stage of the operation, I desist for an hour or two, and resume it again and again, the moment the state of the pulse indicates the safety of the measure." 264.

While using the lancet, Dr. Campbell directed the abdomen to be fomented. The utility of this measure is almost universally allowed. It is grateful to the patient, relieves pain, excites perspiration, and draws the circulation and excitement to the surface, thus lessening the internal plethora of the vessels and irritation of the nervous system of the serous membranes. Professor Hamilton recommends the abdomen to be fomented with hot vinegar; and some practitioners add stimulants to the hot water. We believe there is nothing better than the old form of decoction of poppy heads and chamomile flowers. Some continental practitioners have recommended injections of warm water to be thrown up the vagina, and Dr. Campbell thinks such a measure might be useful; but it has the inconvenience of wetting the bed, without great care. Dr. Mackintosh seems to doubt the propriety of keeping warm fomentations long to the abdomen, lest they should, by their heat, keep up the determination of blood to that quarter. We believe this fear is groundless. Wherever there is much pain or irritation attending an inflammation, warm fomentions will generally be serviceable, even when the phlogosis is external, and the heat consequently applied to the very part inflamed. In peritoneal inflammation there are both pain and irritation; and the heat applied to the external surface relieves these without increasing, but, on the contrary, diminishing internal congestions of blood.

Dr. Campbell's statements respecting local bleeding will be understood by the following extract:—

"Local bleeding is a remedy from which much advantage will be derived; an opinion, which the observations I have had an opportunity of making, in cases attended with uterine effusions, fully confirm. In every instance where we have not succeeded in giving an effectual check to the disease after two or three smart bleedings, we should never neglect the application of a number of leeches to the abdomen.

"From 60 to 100 of these animals should be applied over the surface of this cavity, and as many of them placed in the vicinity of the pudendum, and termination of the round ligaments as possible, because, from the connexion of these points with the uterus, the parts in a state of disease are more likely to be effectually acted on.

The leeches should be re-applied from time to time, according as the pain seems to be determined or renewed towards particular points.* "As the good effects to be expected from their application will depend in a great measure on the subsequent effusion from their bites, it should be carefully promoted. To accomplish this, some have recommended warm cataplasms; others, clothes immersed in warm water. Each, no doubt, has its advantages. The poultices will certainly preserve the bed-clothes, and keep the patient comfortable, by absorbing the effused blood; but the wounds of the leeches will be blocked up sooner by their application, than if the warm compresses had been used. The reiterated application of clothes immersed in warm water, must render the bedding wet and uncomfortable,—so far they are certainly objectionable; but they will assuredly tend to promote the effusion for a longer period than the cataplasms, which is our chief object in applying them." 271.

Dr. Mackintosh recommends the application of from 30 to 100 leeches to the abdomen, after an impression has been made on the system by general bleeding. In our own practice we hesitate to apply more than 24 or 30 leeches at one time. The bites are not so easily stopped as some people imagine, and we have seen alarming debility produced by the protracted issue of blood from leech-bites in some cases of this kind.

In respect to blisters, Dr. Campbell is decidedly against them, and Dr. Mackintosh says he abstained from them in the late epidemic, in consequence of being assured by some of his brethren that they would be of no use. He does not think, however, that he acted wisely in adopting this counsel, and this is our opinion also. We believe that Professor Hamilton advises the application of blisters. We generally apply a very large blister after the leeches have ceased to bleed. It is vain to tell us that a large blister deprives us of the further application of leeches. There are plenty of places for more leeches—the groins, the flanks, and round the circumference of the blister. Dr. Campbell speaks of *sinapisms* and blisters at the same time. If he applied sinapisms to the abdomen, we do not wonder at their producing great irritation and little, if any, good. They are vile and inhuman applications in any disease but those of the comatose class, when there is little sensibility of the surface. They give ten times more pain than blisters—and they do not produce one

---

* "Dr. Frith has stated, that in the Royal Lying-in Hospital, Dublin, leeches were thought preferable to venesection in puerperal fever, and that he has known patients derive much advantage from the application of thirty, forty, or even ninety of them to the abdomen, followed by warm fomentations after their removal."

quarter of the discharge. They never should be applied to the abdomen of man, woman, or child, in an inflammatory complaint.

"In the commencement of the disease," says Dr. Campbell, "free purgation, both in a theoretical and practical point of view, must be considered as highly proper; because at this period, the excitement will be confined to the uterus or peritoneum, or both; and no injury will arise to the intestines from exciting their action by producing increased peristaltic motion, while it is obvious that the secretion from their mucous coat will be increased, and congestion removed.* From the consent of the uterus with the intestines, the secretion from its vessels will also be augmented. When the disease has existed for some time, it is natural to conclude, from the pain becoming general all over the abdominal cavity, and the increased irritability of the stomach, that the intestinal tube is involved in the general derangement, an opinion which dissection has invariably confirmed. In this stage, therefore, I do not think we should be justified in having recourse to active purgation, nor even to an occasional brisk cathartic." 273.

Dr. Mackintosh has also a high opinion of purging. He insists on the necessity of keeping up a constant discharge, "and it is sometimes wonderful to see the copious stools that will come away when the medical attendants have thought it was imposssible that any thing could be in the bowels." He does not approve of drastic purgatives. Castor oil, neutral salts, and the like, are recommended by both our authors. Large enemata are very properly directed by Drs. Campbell and Mackintosh.

"When," says Dr. Campbell, "the intestines are obstinate, a cathartic clyster should be administered every hour, to assist the other purgative medicines. When the bowels have been once opened, the domestic enema, to the amount of lbij. as warm as the individual can comfortably bear it, must be thrown into the rectum every second or third hour, while there is any pain in the abdomen. The enemata will increase the secretion from the vessels terminating on the internal surface of the alimentary canal, and greatly soothe the feelings of the patient, by acting as a fomentation to the internal parts.† In the latter stages of the disease, they should be preferred

* "Dr. Labatt tried the sub. mur. hyd. in doses of ℈j. and ℥ss. as recommended by Dr. Armstrong, and he at first thought its exhibition in such large quantity useful; but he afterwards had reason to be satisfied that it was more beneficial when exhibited in doses of ten or twelve grains in combination with some other purgative, such as jalap.—Armstrong, p. 225."

† "Injections act as fomentations to the uterus, and they should be thrown up frequently, and in large quantity.—MS. of the late Professor Young's Lectures."

to purgatives by the mouth, as being less likely to produce injurious irritation of the intestines." 274.

Dr. Campbell and Dr. Mackintosh are both aware of the great utility to be derived from determining to the surface of the body, and promoting the healthy secretion of the intestinal canal and biliary organ.

"It is of the first consequence," says Dr. Campbell, "to promote general perspiration, with a view that, by determining towards the surface, we may remove local congestion. With this intention, from the moment the cathartic medicines have begun to act, we should exhibit the antimonial oxide in combination with the submuriate of mercury, as directed in the cases. The submuriate, independent of its good effects in promoting the action of the antimony and the cathartics, will also prove beneficial in removing accumulations of the hepatic system." 274.*

Dr. Mackintosh does not point out the means which he employed for the abovementioned purposes. From the *hydrargyro-phobia* which seems to haunt him on all occasions, we suppose he trusted to antimony or ipecacuan alone. If so, we would not advise others to adopt the same practice; for there is but too much tendency to gastric irritability in all abdominal inflammations, and when sickness is once excited, we can seldom allay it afterwards. We should therefore prefer the plan pointed out by Dr. Campbell.

In a foot note Dr. Campbell informs us that Dr. Davis, (of George Street, Hanover Square, London,) after reducing the system by local and general bleeding and purging, orders the abdomen to be covered with a large blister, and digitalis to be administered in powder, in doses of one or two grains every second hour, and very generally with the best effect. It is well known that Professor Hamilton, of Edinburgh, is much attached to digitalis in puerperal fever. Of oil of turpentine, neither of our authors have any good opinion. In tympanitic affections of the abdomen, occurring in this disease, it was found very useful by that able physician, Dr.

* Dr. Vandeuzande, Physician to the Civil Hospital of Antwerp, has lately published a volume on puerperal fever, or puerperal peritonitis, in which he details his almost uniform success in this disease since the year 1808, by pursuing the plan of calomel and opium, first recommended in pulmonic inflammation by our countryman Dr. Hamilton, and since very extensively employed by our tropical practitioners, in fevers and internal inflammations. He asserts, that the cure of puerperal fever was certain, so soon as the salivary glands became affected. He appeals to the testimony of all the pupils and attendants of Antwerp Hospital for the truth of his statements.—*See the Quarterly Journal of Foreign Medicine, &c. No.* 17, p. 78-9.

Labatt, of Dublin. Of Dr. Payne's observations we not again take notice.

Of opium Dr. Campbell scarcely speaks at all; and except an occasional anodyne diaphoretic draught at bed time, in some particular cases, we believe he has never employed that medicine. This we suspect is a defect in his methodus medendi. We have administered it in very large doses after a powerful impression had been made on the system by blood-letting, with very good effects; and we have reason to know that this is the practice generally pursued by Dr. Armstrong at present. Dr. Mackintosh is decidedly favourable to this plan.

"It has already," says Dr. Mackintosh, "been stated, that I have always placed a great deal of dependence on opium, in the cure of inflammatory affections. I used to prescribe one or two grains of solid opium, or from 60 to 100 drops of laudanum, half the previous dose to be repeated every three hours till the full effect was produced. Experience has long ago convinced me, that the apparent want of efficacy of this useful drug, (as well as of many others,) proceeds entirely from its being administered in insufficient doses, and repeated at too long intervals. In inflammatory affections, there is more to be done than the mere abstraction of blood, using purgatives, and calling in to our assistance a strict anti-phlogistic regimen. Nervous irritation alone, if long continued, will undoubtedly produce inflammation; but how much more will it tend to reproduce inflammatory action recently, or perhaps, not yet entirely subdued. It has been proved by experience, that there exists, after child-birth, an increased state of irritability, to allay which, in puerperal fever, I think of more vital consequence, than in any other diseased state of the system." 273.

Dr. Mackintosh found Mr. Batley's liquor opii sedativus a valuable medicine. He begins with a dose of fifteen or twenty drops, and it is repeated in doses of ten drops every hour till relief be obtained.

It is hardly necessary, after the pathology and treatment sketched out here; to say any thing respecting diet and regimen during the fever and convalescence. It is evident that great abstemiousness must be the rule of conduct.

Both our authors take some short notice of what has been termed by Dr. Armstrong the congestive form of puerperal fever, which appears to us to depend entirely on want of power in the system to establish reaction. In some of the unwholesome countries of the earth it is not uncommon for men to die in the cold stage of ague, thus exhibiting exquisite specimens of congestive disease. When congestion takes place in any fever, it is just a predominance of the cold over the hot stages—where they are disproportionately blended, instead of succeeding each other—where one part is in a state of excitement, and another, or many others, in a state of torpor or venous turgescence. These cases are, of course, far more dangerous, and more difficult to manage than those of pure excitement. Dr. Campbell only met with one instance of this kind, and speaks hesitatingly of the treatment, as not founded on personal experience. The following extract presents all that is known on the subject.

"In the treatment of this affection, the great and continual depression of the living powers from the moment it is ushered in, marked by the diminution of the temperature of the body, and the retrocession of the blood from the surface, as well as the rapid and destructive progress of the disease, would seem to suggest more than ordinary efforts on the part of the practitioner to support the powers of the system. At the same time, since the examination of the body after death demonstrates such extensive and decided evidences of congestion in the vessels of the large cavities, every attempt should be made to remove this accumulation of blood, by equalizing the circulating mass, and determining towards the surface.

"Whenever a practitioner is called to a puerperal patient after a paroxysm of rigors, and discovers that its effects have been too permanent, producing an unusually weak, slow, and perhaps an irregular pulse, with great prostration of strength, pallid and collapsed countenance, with coldness of the whole body, but more especially of the extremities, such steps should immediately be adopted as are likely to cause a re-action of the vascular system. With this view, the patient should, with the least possible delay, be placed in the warm bath; or, if she cannot be removed from bed, we should attempt to restore the heat of the body, by covering her with a succession of blankets wrung out of hot water, while she is at the same time to be surrounded with bottles, or bladders, containing hot water; hot irons, or hot bricks, may also be successfully employed for the same purpose. Frictions, with ardent spirits, or with the aq. amon. might prove beneficial; and a cautious attempt must be made to recruit the living powers, by the internal exhibition of some diffusible stimuli—either sulphuric ether or brandy-punch, given in suitable proportions at proper intervals until the temperature of the body be somewhat restored; or camphor, in large doses, may be administered with the same intention.

"In consequence of the blood having receded from the surface, there must be a great accumulation of it in the large veins immediately connected with the heart, in common with other venous trunks; which must not only interrupt the transit of the venous blood from more remote parts towards the right auricle, but even obstruct the immediate action of the heart, an organ, already somewhat paralyzed by the powerful shock which the system has received.

"From this oppressed state of the heart, we might from theory be disposed to subtract blood from the general system, with the intention of relieving this organ; but were we tempted to have recourse to this practice before re-action has commenced, I doubt not but it would speedily determine the fate of the patient. If bleeding, therefore, is ever to be tried, it must be with great caution. When we have succeeded in restoring the action of the heart and arteries, and temperature of the body to some extent, the patient must then be treated according to the symptoms which afterwards present themselves. In these cases, it is scarcely necessary to state, that very little can be effected except at the commencement; and even then, I must agree with Dr. Armstrong in thinking, that the results of our practice, in many instances, are only calculated to throw a stigma on the resources of our art."* 283.

In the few cases of this kind whch Dr. Mackintosh saw, the time for treatment was gone by, and he was only left "a silent witness to the speedy termination of life." From analogy with other congestive diseases, he would place the patient in a bath of 112°, and "if possible, open a vein while immersed." We would be more inclined to wait till re-action came on by the bath and diffusible stimuli—and then to bleed according to the degree of the superinduced excitement.

Dr. Campbell closes the original part of his work with many judicious observations on the prevention of puerperal fever, and, indeed, of puerperal diseases in general, which are well worthy the attention of the junior branches of the profession. He then introduces tables indicative of the state of the weather during the prevalence of the late epidemic, concluding with an appendix, containing a republication of Dr. Gordon's Treatise on the Epidemic Puerperal Fever of Aberdeen, originally published in 1795, and now out of print and very scarce.

Dr. Mackintosh concludes his work with an analytic sketch of the practice of Hulme, and a short review of the cases of Leake, Gordon, and Hey, principally with the view of shewing the failure of bleeding, from not being boldly and confidently followed up; and the superior success of that practice, in the same hands, when early and copiously employed. Dr. Mackintosh has, also, added an appendix, containing eight pages of strictures on Mr. Moir's case of puerperal fever, on which we made a short commentary in our last number. Dr. M. has come to almost exactly the same conclusions as ourselves—and after all, we think, the case was hardly worth so much criticism.

In the endeavour to present our readers with a copious view of the etiological, pathological, and therapeutical matters contained in these two important volumes, we have left

* Campbell, p. 282-3.

ourselves no space for extracting any of the numerous and highly interesting cases and dissections detailed in the works before us, especially in Dr. Campbell's publication, which contains the histories of no less than *forty-eight* cases, with many dissections. We never remember to have seen such a strict coincidence of opinion and practice between any two cotemporary medical writers, as Drs. Campbell and Mackintosh have here evinced—a circumstance that not a little increases our confidence in the accuracy and fidelity of both writers, and in the truth of the doctrines and practices they embrace. It cannot be necessary to inform our readers, that we are attached to the views which are here advocated;—not from theory, but from observation of facts, and from a pretty extensive field of pathological investigation. We cannot, therefore, but view with concern, an attempt in the northern metropolis to revive exploded notions and antiquated pathology, with all the departed ghosts of debility and putrescency, which we had hoped were laid in the tomb of the Capulets. The publications under review, are well calculated to counteract these anile superstitions, so long and so often stained with the blood of their victims; and, therefore, we have been anxious to give as much scope and publicity as possible to the contents of the volumes before us. The only objections we have to urge, have already been hinted at in the course of this article—namely, the non-admission, on the part of our authors, of the modifying influence of epidemic constitutions. Dr. Mackintosh does not even allude to this subject, and Dr. Campbell concludes that the epidemic puerperal fever only differs from the sporadic, in requiring more decided measures of depletion. We are quite ready to admit that the symptoms and dissections of the late epidemic of Edinburgh sanction this conclusion, *as far as that epidemic was concerned*, and that the authors in question were perfectly justifiable in the depletive measures they so ably put in force. But still we are unwilling to admit the *general principle*, that all epidemic fevers are of the same character, and require the same treatment. There is another point in which we do not quite coincide with our authors—*their denial of contagion* in puerperal fever under certain circumstances. We think there is good proof on record that a contagious character is occasionally added to its other bad properties; and as this belief is not calculated to do any harm that we know of, but some good, in as much as it will lessen the number of intrusive visitors, we think too much scepticism is rather reprehensible.

In fine, we are compelled, by our duty to the public, to state our preference of Dr. Campbell's work, as containing more dense practical matter, and less speculation, than the work of Dr. Mackintosh. The latter work, at the same time, we have no hesitation in denominating a very able production, and that it only suffers by comparison with a cotemporary which appears to be erected on a more extensive experience of the epidemic in question, and to be constructed with more care and less precipitation. We infer, that it was constructed with less haste, because it is far more systematic, and far more clearly arranged. On this, and many other accounts, it is better adapted for the end which it is designed to attain, than the work of Dr. Mackintosh. We are sorry, indeed, that these two deserving practitioners, who were frequently in attendance on the same patients, and whose views of the complaint so nearly harmonized, should not have coalesced in one work, as is often done on the Continent, and thus saved the public the purchase of two instead of one publication. We trust that the public will award to us the merit of having candidly and impartially analysed the productions before us; and to them we appeal, also, for the justice of the decision we have ventured to pronounce on their comparative merits.

P.S. Just as the last sheet of this article was going to press, we received a pamphlet, entitled "Notes on Dr. Mackintosh's Treatise on Puerperal Fever, by James Moir, Surgeon." It appears to be a tissue of the most slanderous abuse and libellous defamation of Drs. Mackintosh and Campbell, that we ever had the misery of perusing! We cannot sully our pages with any notice of so malevolent and scandalous a production. As a great portion of it is taken up with the defence of Professor Hamilton, and as several of that gentleman's communications to the author are therein printed, we have little doubt in our own minds, of the *real source* whence the pamphlet issues. It requires, and deserves no other argument than the *argumentum baculinum*, which it would, long ere this, have received, had it been put forth in "Paddy's land" instead of "Auld Reekie." If the gentlemen thus traduced can tamely put up with such insults, it must be for the purpose, we should imagine, of prosecuting the infatuated and enfuriated author for a libel. We had hardly supposed that such rancorous personalities would, at this time of day, have issued from the medical press; but Edinburgh is famous for these productions—and the more shame for her!

